# Comparative Genomic Hybridization Analysis of *Yersinia enterocolitica* and *Yersinia pseudotuberculosis* Identifies Genetic Traits to Elucidate Their Different Ecologies

**DOI:** 10.1155/2015/760494

**Published:** 2015-10-28

**Authors:** Kaisa Jaakkola, Panu Somervuo, Hannu Korkeala

**Affiliations:** Department of Food Hygiene and Environmental Health, University of Helsinki, P.O. Box 66, 00014 Helsinki, Finland

## Abstract

Enteropathogenic *Yersinia enterocolitica* and *Yersinia pseudotuberculosis* are both etiological agents for intestinal infection known as yersiniosis, but their epidemiology and ecology bear many differences. Swine are the only known reservoir for *Y. enterocolitica* 4/O:3 strains, which are the most common cause of human disease, while *Y. pseudotuberculosis* has been isolated from a variety of sources, including vegetables and wild animals. Infections caused by *Y. enterocolitica* mainly originate from swine, but fresh produce has been the source for widespread *Y. pseudotuberculosis* outbreaks within recent decades. A comparative genomic hybridization analysis with a DNA microarray based on three *Yersinia enterocolitica* and four *Yersinia pseudotuberculosis* genomes was conducted to shed light on the genomic differences between enteropathogenic *Yersinia*. The hybridization results identified *Y. pseudotuberculosis* strains to carry operons linked with the uptake and utilization of substances not found in living animal tissues but present in soil, plants, and rotting flesh. *Y. pseudotuberculosis* also harbors a selection of type VI secretion systems targeting other bacteria and eukaryotic cells. These genetic traits are not found in *Y. enterocolitica*, and it appears that while *Y. pseudotuberculosis* has many tools beneficial for survival in varied environments, the *Y. enterocolitica* genome is more streamlined and adapted to their preferred animal reservoir.

## 1. Introduction

Enteropathogenic* Yersinia* is the third most common cause of bacterial enteritis in European countries, even though a statistically significant decreasing 5-year trend in yersiniosis cases has been reported in the EU [1]. Infection is usually foodborne, with symptoms ranging from self-limiting diarrhea to reactive arthritis or erythema nodosum [2].* Yersinia* are Gram-negative rods belonging to Enterobacteriaceae. Enteropathogenic* Yersinia* diverged around 41–185 million years ago, while the third human pathogenic species of* Yersinia* genus, the infamous* Yersinia pestis*, is a relatively recent clone of* Yersinia pseudotuberculosis *[3]. The evolution of enteropathogenic* Yersinia* is thought to have included multiple distinct ecological specializations that have separated the pathogenic strains from environmental, nonpathogenic lineages. This current hypothesis of parallel evolution [4] rejects the previous one suggesting that all pathogenic* Yersinia* species share a common pathogenic ancestor [5].

Enteropathogenic* Yersinia enterocolitica* and* Y. pseudotuberculosis* cause similar infections, but their epidemiology and ecology appear to differ in many aspects. Both* Y. enterocolitica *and* Y. pseudotuberculosis* have been isolated from swine or pork, and yersiniosis has been associated with the consumption of uncooked pork [6–8]. Traditionally, most cases of yersiniosis are thought to occur sporadically, and cases caused by* Y. enterocolitica* are mostly associated with pork products [7, 9–11]. In rare cases, the source of human infection has been traced back, for example, to milk, poultry meat, and ready-to-eat salad [12–14]. Within recent decades, several widespread outbreaks caused by* Y. pseudotuberculosis* have been reported in Finland [15–18]. The sources of the infections have been traced back to fresh produce, such as iceberg lettuce [15] and grated carrots [18–20]. The epidemic strain involved in an outbreak caused by raw carrots was also recovered from the field and production line [19]. The genetic traits underlying the observed epidemiological differences remain poorly understood.

Research has shown that the prevalence of* Y. enterocolitica* in swine is notably higher than that of* Y. pseudotuberculosis*, and swine are the only reservoir from which* Y. enterocolitica* 4/O:3 strains have regularly been isolated [2, 25–27]. The most common cause of* Y. enterocolitica* infection in humans in Africa, Europe, Japan, and Canada is* Y. enterocolitica *4/O:3. Bioserotype 4/O:3 is considered as an emerging pathogen, while the prevalence of the second most common pathogenic bioserotype,* Y. enterocolitica *1B/O:8, is diminishing [2, 10, 28, 29]. Extensive research has been carried out to uncover the virulence factors of* Y. enterocolitica* and its different serotypes [30–35], and the virulence factors explaining the swine specificity of* Y. enterocolitica *serotype O:3 were recently identified [22, 36]. The differences in virulence gene expression patterns alter the surface adhesion properties and cytokine production profiles of O:3 strains and thus probably permit the asymptomatic infection and long-term colonization of the nasopharynx and intestine of swine [22].

Pathogenic and nonpathogenic* Y. enterocolitica *strains are frequently found from wildlife samples such as water fowl and hares, but pathogenic strains have rarely been isolated from soil or water [2, 8, 37].* Y. pseudotuberculosis* strains have been isolated from a variety of sources, including fresh vegetables and wild animals, and contrary to* Y. enterocolitica*, all strains are considered pathogenic [8, 15–17, 38]. Despite the frequent presence of* Y. pseudotuberculosis* in environmental samples, its reservoir is considered to be wildlife [38, 39].

A comparative genomic hybridization (CGH) analysis with a DNA microarray based on three* Y. enterocolitica *and four* Y. pseudotuberculosis* genomes was conducted to shed light on the genetic traits and ecological specializations explaining the epidemiological differences between enteropathogenic* Yersinia*. Our hypothesis was that the genomes would contain operons elucidating the ways in which* Y. enterocolitica* has adapted to its mammal hosts and the ecology of* Y. pseudotuberculosis.* The strains hybridized on the microarray were isolated from human, animal, and environmental samples.

The hybridization results revealed that* Y. pseudotuberculosis *strainscarry many operons linked with the uptake of carbohydrates and use of aromatic substances that are absent from* Y. enterocolitica*. Phenolic compounds, polyamines, myoinositol, and aliphatic sulfonates are all substrates that are not commonly present in living animal tissue, but more abundant in plants and the soil environment.* Y. pseudotuberculosis *also harbors an array of different types of type VI secretion systems (T6SSs), in contrast to just one found in the* Y. enterocolitica *genome. These T6SSs are likely to provide defense against other bacteria and single-celled organisms in the environment.

## 2. Materials and Methods

### 2.1. Bacterial Strains for Hybridization

A total of 60* Y. enterocolitica *and 38* Y. pseudotuberculosis *strains isolated from a variety of geographic locations and sources were used in this study (Table S1 in Supplementary Material available online at http://dx.doi.org/10.1155/2015/760494). The strains were selected to represent the different biotypes and serotypes of enteropathogenic* Yersinia*, and also included were three strains (*Y. enterocolitica* subsp.* enterocolitica *8081,* Y. enterocolitica* subsp.* palearctica *Y11 [DSM 13030], and* Y. pseudotuberculosis* IP32953) used in the microarray design. These three strains were used as reference strains and as positive hybridization controls. The reference strains and one additional strain were hybridized in quadruplicate to assess the reproducibility of the hybridizations. The reference strains produced positive hybridization signals with 99.4–99.9% of the probes designed to hybridize with their sequences.

In total, 41 strains represented the most common pathogenic* Y. enterocolitica* bioserotype 4/O:3. The majority (79/98) of the strains had been isolated from swine or from swine slaughterhouses. The rest of the strains (*n* = 19) were isolated from human patients, wild birds, and other animals.

### 2.2. DNA Microarrays

The DNA microarrays were designed based on seven genomes and 14 plasmid sequences (Table S2) obtained from the NCBI database. 29,786 sequences were clustered into 11,564 gene groups by Cd-hit-est [41]. The threshold value of identity was set to 95% with minimum alignment of at least 80% of the longer sequence. Stringent clustering parameters were chosen to avoid problems with uncomplimentary probes in the probe design. With these parameters, the number of unique sequences (gene groups containing a sole sequence) amounted to 3747.

One 45–60-mer probe was designed for each gene group (*n* = 11,564). Thirteen gene groups containing a total of 14 sequences were excluded from the probe design because of redundancy. The longest gene sequences were over 10,000 bases long (*n* = 11), and for these a tiling method was used for the design of extra probes (10 per sequence). All probes were designed using Agilent Technologies Gene Expression Probe Design. Each of the eight subarrays of Agilent 8*∗*15 K custom arrays (Agilent, Santa Clara, CA, USA) contained an equal set of 11,661 probes.

### 2.3. Hybridization and Washes

Genomic DNA was isolated using a method described by Pitcher et al. [42]. A total of 500 ng of genomic DNA from each* Yersinia *strain was fluorescently labeled with the BioPrime ArrayCGH labeling module (Invitrogen, Carlsbad, CA, USA) using either Cy3 or Cy5 (GE Healthcare, Buckinghamshire, UK). For each hybridization, one Cy3-labeled and one Cy5-labeled DNA sample were combined. The mixture was purified with a DNA purification kit (QIAquick PCR Purification Kit, Qiagen, Hilden, Germany) according to the manufacturer's instructions. The concentration of DNA and the incorporation of the dye were checked with the Nanodrop device (Nanodrop Technologies, Wilmington, MA, USA) before and after labeling. The differently labeled DNA sample pairs to be hybridized into one of the eight subarrays on each array slide were randomly selected.

A volume of 2.2 *μ*L salmon sperm DNA (1 mg/mL) was added to 17.8 *μ*L of labeled combined sample solution, and the mixture was heated at 95°C for 2 minutes for denaturation. A volume of 5 *μ*L of 10x blocking agent (Agilent) and 25 *μ*L of 2xGE (HI-RPI) hybridization buffer (Agilent) was added. A total of 45 *μ*L of the solution was hybridized on each microarray at 65°C for 16 hours. The arrays were washed twice for 1 minute with Wash Buffer 1 (Agilent) and then for 1 minute with prewarmed Wash Buffer 2 (Agilent).

### 2.4. Scanning and Data Analysis

The CGH data analysis followed the routines set by Lindström et al. [43] and Lahti et al. [44]. The slides were scanned (Axon Genepix Autoloader 4200 AL, Molecular devices Inc., Sunnyvale, California, USA) with a resolution of 5 *μ*m. Images were processed and manually checked using GenePix Pro 6.0/6.1 software. For data analysis, R software and the LIMMA package were used [40, 45]. For background correction, the normexp algorithm (offset 50) was applied [46].

The distribution of logarithmic signal intensities formed two clear peaks in all hybridizations and a method conforming the positions of these density peaks was used to normalize the hybridization data. Standard normalization methods for microarrays are unsuited for CGH data since the distribution of intensities between different hybridizations cannot be assumed to be thesame. Conforming the positions of density peaks is based on an assumptionthat all hybridizations exhibit high densities of both positive andnegative hybridization signals but does not alter the distribution patternof intensities. By positive and negative hybridization signals, we here mean signals representing present and absent/divergent genes, that is, high and low intensity signals, respectively. Visualization and clustering of data were conducted using MEV [47].

The distribution of logarithmic signal intensities was also used to set a threshold between the intensity peaks (lowest point of density) separately for each hybridization. This threshold was used to classify the probes and the corresponding genes as present, absent, or diverged in each strain. Intensity values of the threshold value ±0.3 were classified as diverged. The number of probes classified as “diverged” varied from 0 to 2 in all hybridizations, and these probes were considered as absent in further data analysis.

The data discussed in this paper are compliant with the MIAME guidelines and were deposited in NCBI's Gene Expression Omnibus and are accessible through GEO Series accession number GSE67565 (http://www.ncbi.nlm.nih.gov/geo/query/acc.cgi?acc=GSE67565).

### 2.5. Phylogenetic Analysis of Type VI Secretion System Sequences

Phylogenetic analysis similar to analysis described by Schwarz et al. [48] was performed on VipA sequences stored in the NCBI database and annotated to TIGR category COG3516 (*n* = 195). This TIGR sequence pool was supplemented by sequences annotated as VipA in* Y. pseudotuberculosis* IP32953 (*n* = 4),* B. thailandensis* (*n* = 5),* P. aeruginosa* (*n* = 1),* Y. enterocolitica *Y11 (*n* = 1), and* Y. enterocolitica *8081 (*n* = 1). In total, 206 VipA sequences were aligned using MUSCLE [49] and the resulting alignment was visualized with BioNJ [50].

### 2.6. Orthologous Genes

Reciprocal Blast searches were performed to identify the bidirectional best hits between the genomes used in microarray design. Any bidirectional best hit identified was assumed to represent an orthologous gene pair [51]. Information on orthologous gene pairs was used as an aid in the interpretation of the microarray data.

## 3. Results

**Figure 1:**
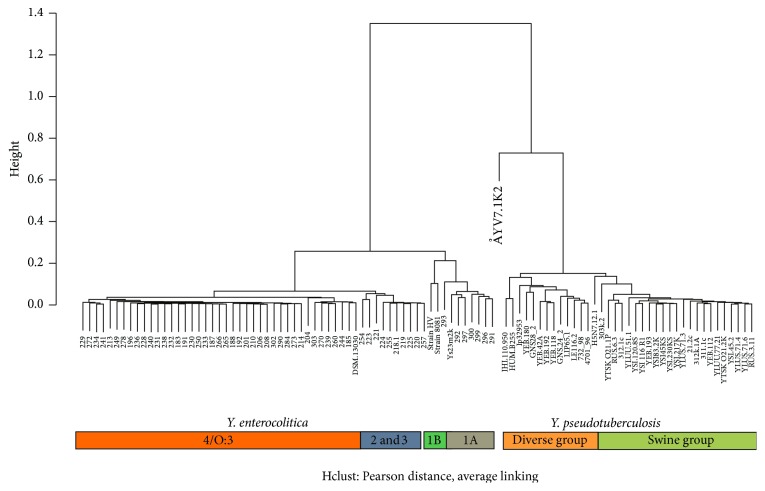
*Yersinia enterocolitica *and* Yersinia pseudotuberculosis* strains group into two distinct clusters and* Y. enterocolitica *strains belonging to four different biotypes form distinct subclusters within the* Y. enterocolitica *group. The majority of* Y. pseudotuberculosis* strains obtained from swine samples cluster separately (“swine group”) from strains obtained from human and wildlife samples (“diverse group”). The diverse group of* Y. pseudotuberculosis* also includes some strains isolated from English swine. Hierarchical clustering was constructed using R [40].

CGH analysis of 60* Y. enterocolitica* and 38* Y. pseudotuberculosis* strains was conducted with a DNA microarray based on three* Y. enterocolitica* and four* Y. pseudotuberculosis* genomes to shed light on genomic differences between enteropathogenic* Yersinia*.* Y. enterocolitica *strains and* Y. pseudotuberculosis *strains grouped into two distinct clusters (Figure 1).* Y. enterocolitica *strains belonging to four different biotypes formed distinct subclusters within the* Y. enterocolitica *group. Strains belonging to* Y. enterocolitica* biotype 2 or 3 (*n* = 10) clustered together (Figure 1). The distance between strains, based on Pearson's correlation on a scale from 0 to 2 (0 indicating identical samples), was 0.25 between* Y. enterocolitica *biotypes 2–4 and biotypes 1A and 1B. Within the* Y. pseudotuberculosis* group, the distance was 0.15. The distance between* Y. enterocolitica* and* Y. pseudotuberculosis *group was 1.36.

All hybridized strains produced positive signals on 459 probes, which is the equivalent of 320–360 genes depending on reference genome used. This means that around 8% of the genome is fully conserved across the two species. In the seven genomes used in array design, the core genome based on bidirectional best hits contained 2772 sequences, implying that 68–76% of genes in each sequenced genome have orthologous equivalents in the rest.

Comparing the 320 shared probes in hybridization results and 2772 in orthologous gene pairs, it becomes clear just how sensitive microarray hybridization is as a research method. Out of the 3547 probes (gene clusters) deemed present in all* Y. enterocolitica* strains, 1130 did not show a positive hybridization signal in any* Y. pseudotuberculosis* strain and were thus considered specific for* Y. enterocolitica *(Table S3). When these 1130 gene clusters were further pruned using the information on orthologous gene pairs, only 448 gene clusters remained truly specific for* Y. enterocolitica*. Similarly, in the* Y. pseudotuberculosis* group, 906 gene clusters were deemed conserved and specific (Table S3).* Y. enterocolitica* bioserotype 4/O:3 strains (*n* = 42) shared 51 gene clusters that were only extant in strains of this bioserotype. This represents around 1% of genes in the sequenced* Y. enterocolitica* 4/O:3 genome Y11.

*Y. pseudotuberculosis* strains shared three large operons coding type VI secretion systems that were missing from* Y. enterocolitica* strains.* Y. pseudotuberculosis* strains also shared a variety of gene clusters that based on their annotation are likely to be involved in the use and/or uptake of various substrates, including phenolic compounds, rhamnose, xylose, myoinositol, opines/polyamines, and aliphatic sulfonates (Table 1).* Y. enterocolitica *strains share six ATP-binding cassette (ABC) transporters and seven phosphotransferase systems (PTS) that are all absent from* Y. pseudotuberculosis* strains (Table 1). In addition, the* Y. enterocolitica *strains, excluding the highly virulent 1B strains, carry an operon involved in the utilization of N-acetylgalactosamine.* Y. pseudotuberculosis* strains share 18 ABC transporters and 2 PTS transporters that are absent from* Y. enterocolitica* strains.

**Figure 2:**
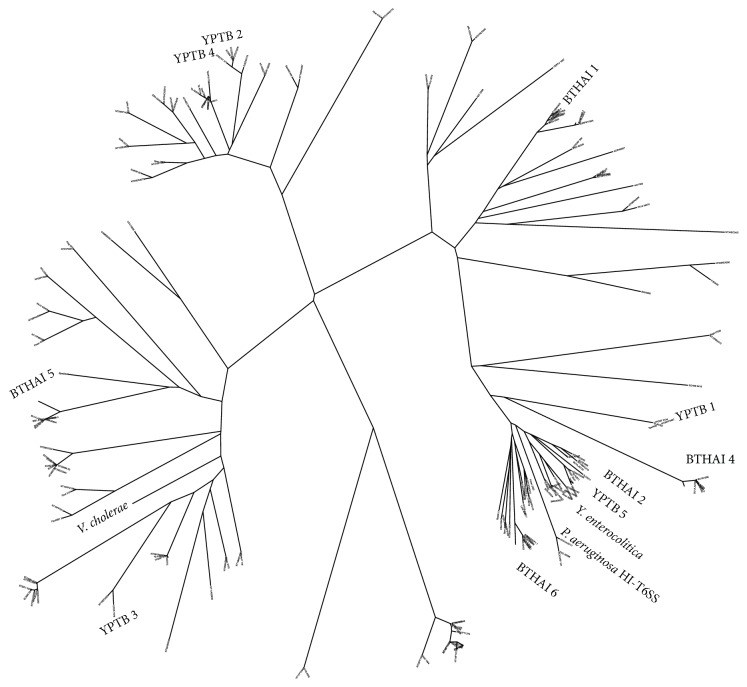
Phylogenetic relationships of type VI secretion systems (T6SSs) in* Yersinia pseudotuberculosis* and T6SSs of other species were compared to evaluate the different types of T6SS. VipA sequences were used to represent T6SS and the alignment of 206 VipA proteins is shown here as an unrooted phylogenetic tree visualized by BioNJ. Type VI secretion systems of* Y. pseudotuberculosis *named YPTB 1–5 belong to three distinct branches of T6SSs. T6SSs of* Vibrio cholerae*,* Pseudomonas aeruginosa*, and* Burkholderia thailandensis* (BTHAI 1–6) are marked on the branches of the phylogenetic tree and the one VipA/T6SS present in* Y. enterocolitica* is also shown.

Phylogenetic analysis of the different T6SSs shows that three distinct types of T6SS are present in* Y. pseudotuberculosis* (Figure 2). One type present in two copies in* Y. pseudotuberculosis* (CAH19881.1, CAH21904.1 in* Y. pseudotuberculosis* IP32953) is present in one copy in* Y. enterocolitica* genomes (CAL12724.1 in strain 8081) and bears strong similarity to several T6SSs found in other bacteria. These include H1-T6SS found in* Pseudomonas aeruginosa* and four T6SSs found in* Burkholderia thailandensis*. Copies of the second type of T6SS (CAH20722.1 and CAH22490.1 in* Y. pseudotuberculosis* IP32953) clustered together with uncharacterized T6SSs found in* Y. pestis* (Figure 2). The function of T6SSs belonging to this type is unknown. The third distinct type of T6SS identified in* Y. pseudotuberculosis* genomes (CAH22876.1 in IP32953) shared strong similarity with the T6SSs of* Vibrio cholerae* and* B. thailandensis*, which are both considered to have cytotoxic effects against unicellular organisms and macrophages.

**Figure 3:**
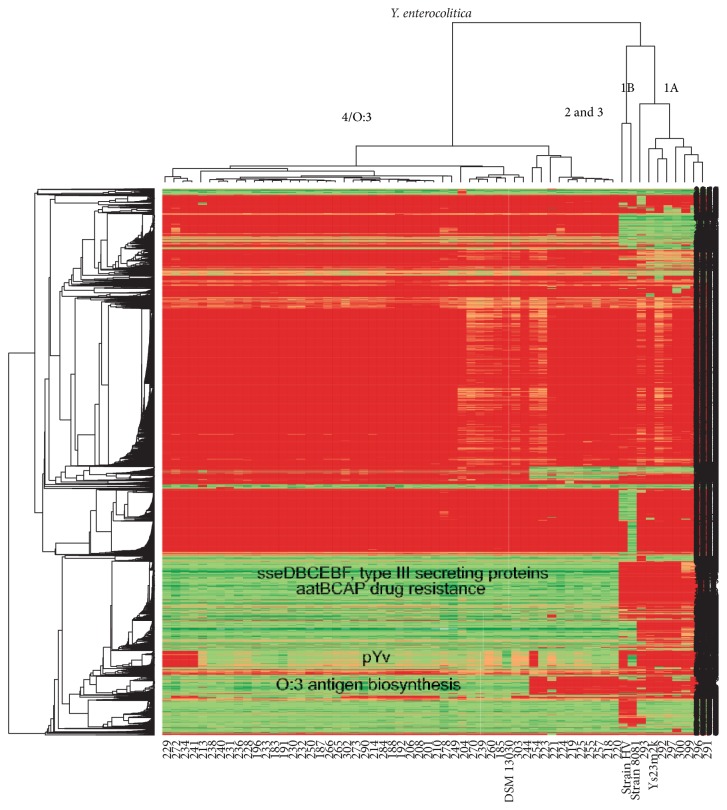
A heatmap presentation of* Yersinia enterocolitica* hybridization results produced with R. Biotypes cluster separately and major gene clusters differing between different biotypes are shown. A green color signifies that the gene is present in the given strain.

Relatively few gene cluster differences were observed between the hybridization results of different* Y. enterocolitica *strains (Figure 3). These included an operon coding for type III secretion system shared by low-pathogenic* Y. enterocolitica*, genes involved in drug resistance, and the operon coding from O:3 antigen. Many of the other differences are annotated as putative phages or flagellar components.

The majority of* Y. pseudotuberculosis* strains obtained from swine samples (“swine group” in Figure 1) in Finland, Sweden, Estonia, Russia, England, and Belgium (*n* = 23) clustered separately from human and wildlife samples (“diverse group” in Figure 1). Five of the 11* Y. pseudotuberculosis *strains isolated from English pigs clustered together with the human and wildlife samples.

## 4. Discussion

*Y. enterocolitica *and* Y. pseudotuberculosis* strains grouped into two distinct clusters, and* Y. enterocolitica *strains belonging to four different biotypes formed distinct subclusters within the* Y. enterocolitica *group. On the gene level, the most interesting differences between* Y. enterocolitica *and* Y. pseudotuberculosis *strains included genes involved in T6SSs, the catabolism of phenolic compounds, and the transport of many carbohydrates (rhamnose, fructose, ribose, myoinositol, and xylose) and other compounds (aliphatic sulfonates, opines) (Table 1).

T6SS forms a needle-like injectisome between the bacterial cell and the target cell [52]. First described under ten years ago, T6SS is now one of the most common large specialized secretion systems found in over 120 bacteria [48, 53]. T6SSs were first considered as virulence factors, but their abundance in nonpathogenic bacteria and further studies have suggested that most of these systems play a role in interbacterial interaction and defense against competitive bacteria and unicellular organisms [54]. The mechanism requires 15 conserved genes and direct contact with other cells and is thus thought to be especially useful in the stationary growth phase [52, 55, 56]. It is notable that diverse collections of T6SSs have been reported in many environmental bacteria with facultative pathogenic potential, such as* Pseudomonas aeruginosa*,* Burkholderia mallei*, and* Burkholderia pseudomallei*, as well as in bacteria with multiple hosts and the ability to survive in diverse environmental conditions (*Y. pestis*,* V. cholerae*) [53, 57–60].* Y. pseudotuberculosis* is also considered a facultative pathogen with multiple host species and able to persist in the environment. The genome of* Y. pseudotuberculosis* has four conserved systems and one smaller, perhaps partial system [55]. Only one of these systems is shared with* Y. enterocolitica*. The loci coding VipA protein is used to indicate the location of each T6SS. Two of the* Y. pseudotuberculosis* T6SSs (CAH19881.1, CAH21904.1 in IP32953) and the solitary T6SS in* Y. enterocolitica *(CAL12724.1 in strain 8081) group in phylogenetic analysis together with* B. thailandensis* T6SS (BTHAI-1) and H1-T6SS of* P. aeruginosa.* The latter two have been reported to target other bacteria and give some competitive advantage to the bacterium itself  [48, 56]. Having this mechanism could enhance the growth of* Y. pseudotuberculosis* when other bacteria are present on a shared growth surface. Another T6SS (CAH22876.1 in IP32953) is similar to the T6SSs in* V. cholerae* and* B. thailandensis*, which are described as being cytotoxic against single-celled organisms and macrophages [48, 53, 61]. T6SSs like this are beneficial against protists living in the soil and water environment but are also possible pathogenicity factors. To better understand the function of each T6SS of* Y. pseudotuberculosis*,* in vivo* studies are required. Epidemiologically, T6SSs could probably help* Y. pseudotuberculosis* to survive and multiply in such ecological niches in the environment from which it could easily end up as a contaminant of the food chain. The lack of T6SSs in* Y. enterocolitica *implies that the organism in its current ecological niche has no need for them. The lack of T6SSs might actually be beneficial for the organism, as a T6SS with cytotoxic effects against the macrophages of the mammal host might encumber the invasion and survival of* Y. enterocolitica* cells.

*Y. pseudotuberculosis* strains also carry a variety of gene clusters involved in the uptake and/or utilization of various substrates (Table 1). The Hpa operon (CAH20875.1–CAH20884.1 in IP32953), also known as the 4-hydroxyphenylacetate degrading operon, is involved in the catabolism of phenolic and aromatic compounds [62, 63]. Based on database queries, Hpa sequences in* Y. pseudotuberculosis *are homologous to those in* E. coli* and* Salmonella*. Phenols are products of plant secondary metabolism and often have bactericidal effects. Phenols are widely present in soil and the water environment, but their abundance in the intestines of animals has also been suggested [62, 63]. Interestingly, the hpa genes are expressed in* Salmonella enterica* serovar Typhimurium cells during the infection in swine, and it has been suggested that the operon is somehow beneficial for enteropathogenic bacteria [64]. Evidently, the lack of an Hpa operon does not seem to hinder the prevalence of* Y. enterocolitica *in swine.

Both* Y. enterocolitica *and* Y. pseudotuberculosis *have many transport and uptake systems that are not present in other species.* Y. enterocolitica *strains share six ABC transporters and seven PTS transporters that are all absent from* Y. pseudotuberculosis* strains. Putative substrates for these systems include iron and metallic ions, glycosides, and lactose/cellobiose. Interestingly, one putative urea ABC transporter was noted as present in all other* Y. enterocolitica *strains, but absent from reference strain 8081. This highlights the benefits of having multiple reference strains.

*Y. enterocolitica *strains shared notably fewer specific genes (*n* = 448) between them than* Y. pseudotuberculosis *strains (*n* = 906). This is likely to reflect the greater heterogeneity within* Y. enterocolitica* biotypes and the ecological adaptation of biotypes 2–5 by gene loss and genome decay [4]. Many of these specific genes were involved in transportation.* Y. pseudotuberculosis* strains shared 18 ABC transporters and 2 PTS transporters absent from* Y. enterocolitica*. Putative substrates for these systems include rhamnose, fructose, xylose, myoinositol, iron, aromatic acids, polyamines, sorbitol, sulfonates, and 7 systems for unspecified sugars. The* Y. pseudotuberculosis *genome appears to be equipped with many extra tools for moving substances in and out of its cell compared to the* Y. enterocolitica *genome, which appears more streamlined and likely to have adapted to another ecological niche, such as swine tonsils and gut, where variety in substrate transportation is not required. A recent hypothesis on the evolution of enteropathogenic* Yersinia* assumes that* Yersinia* species have evolved to become more ecologically specific and metabolically more limited to their reservoirs by genome decay and gene loss [4]. An evolutionary path such as this appears plausible, as* Y. enterocolitica* seems better adapted to living in a mammal host and to have lost many genes involved in survival in the environment.

The theory of genome decay and gene loss, however, does not explain the differences between* Y. enterocolitica* biotypes. Lipase activity, hydrolysis of B-glycosides such as salicin and esculin, use of xylose, and indole production are some of the biochemical tests belonging to* Y. enterocolitica *biotyping schema [2]. However, relatively few clusters of genes differentiate pathogenic and nonpathogenic* Y. enterocolitica* strains from each other. Recent results have identified the changes in gene expression patterns for pathogenicity factors explaining the swine specificity of* Y. enterocolitica* 4/O:3 [22]. It seems likely that the adaptation and differences in pathogenicity of* Y. enterocolitica* biotypes are due to point mutations and changes in gene expression rather than gene loss.

*Y. pseudotuberculosis* strains obtained from swine samples mostly clustered separately from human and wildlife samples (Figure 1). Notably, five of the 11* Y. pseudotuberculosis *strains isolated from English swine clustered together with the human and wildlife samples. Niskanen et al. [39] have previously reported on the homogeneity of* Y. pseudotuberculosis* strains isolated from swine samples based on pulsed-field gel electrophoresis analysis. Our results further confirm this finding. Martínez et al. [25–27] noted that the prevalence and diversity of* Y. pseudotuberculosis *strains appears to be higher in English swine than in swine of other European countries. This finding is also supported by the present results, as 5 of the 11 English* Y. pseudotuberculosis *strains showed a marked genetic distance to other swine strains. In these results, no defining gene cluster setting the swine group and diverse group apart could be identified. In this type of study, the results are dependent on the reference strains used. It is important to note that because of the limitations of the method, many genes present in the studied strains might be absent from the reference genomes used and thus from the designed microarray and the further results. It would be interesting to have a wholly sequenced genome from the “swine group” of* Y. pseudotuberculosis *for further research on the differences between these two groups.

The high prevalence of* Y. pseudotuberculosis *in the English pork chain is probably explained by the more available access to outdoors of English swine compared to their continental counterparts. Swine and pork products are not considered to be a notable source of sporadic* Y. pseudotuberculosis *cases, and animals having greater contact with the environment are more likely to have strains of soil and wildlife origins passing through their intestines. This would also explain why some* Y. pseudotuberculosis *not belonging to the “swine group” of* Y. pseudotuberculosis *have been isolated from English swine.

## 5. Conclusions

The hybridization results revealed that* Y. pseudotuberculosis *strains carry many operons linked with the use of carbohydrates and other substrates that are absent from* Y. enterocolitica*. Phenolic compounds, polyamines, myoinositol, and aliphatic sulfonates are all substrates that are not commonly present in living animal tissue but are more abundant in soil and the environment.* Y. pseudotuberculosis *also harbors an array of different type VI secretion systems, in contrast to just one found in the* Y. enterocolitica *genome. Type VI secretion systems target single-celled organisms and other bacteria but are also possible pathogenicity factors. These defense and interaction systems could help* Y. pseudotuberculosis* to survive and multiply in such ecological niches in the environment from which it could easily end up as a contaminant of the food chain.

The* Y. pseudotuberculosis* genome holds many tools, such as type VI secretion systems and transporters for various substrates, which are likely to be beneficial for survival in varied growth environments and multiple host species. By comparison, the genome of* Y. enterocolitica* appears more streamlined and likely to have adapted to a different ecological niche where these survival systems are not needed or beneficial. For* Y. enterocolitica* bioserotype 4/O:3, this niche is with certainty swine.

## Supplementary Material

Supplementary material includes the information of bacterial strains used for hybridization (Table S1), the information of sequences used for microarray design (Table S2) and a comparison of number of gene groups present, specific and unique for Yersinia pseudotuberculosis and Yersinia enterocolitica (Table S3).

## Figures and Tables

**Table 1:** Main differences in gene clusters between enteropathogenic *Yersinia enterocolitica *(YE) and *Yersinia pseudotuberculosis* (YP) strains.

Present in	Absent from	Locus	Gene names	Role	Description and comments
YE	YP	CBY25444.1- CBY25441.1	*aapJQMP*	Transportation	ABC transporter (L-amino acids).

YE	YP	CBY25938.1- CBY25944.1	*sorEMABF*	Transportation	Phosphotransferase system (sorbose) [21].

YE	YP, YE str.* 8081*	CBY25947.1- CBY25953.1	*urtEDCBA*	Transportation	ABC transporter (urea). This copy of operon is absent from strain 8081.

YE	YP	CBY26058.1- CBY26056.1	*aglBA*	Transportation	Phosphotransferase system (alpha-glycosides).

YE	YP	CBY26159.1- CBY26152.1	—	Transportation	ABC transporter (metallic ion).

YE	YP	CBY26547.1- CBY26568.1	*pduVUTONMLKJBA*	Propanediol utilization	1,2-Propanediol utilization [21].

YE	YP	CBY26570.1- CBY26589.1	*cbiGKNQO*	Propanediol utilization	Cobalamin synthesis [21].

YE	YP	CBY26648.1- CBY26640.1	*citXFEDCAB*	Metabolism	Citrate lyase, ability to ferment citrate in anaerobic conditions.

YE	YP	CBY26805.1- CBY26815.1	*rutGEFDCGR*	Nitrogen metabolism	Pyrimidine utilization. Use of pyrimidines as a source of nitrogen in *E. coli*. Genes *rutA* and *rutG* are interrupted in *Y. enterocolitica*.

YE	YP	CBY28018.1- CBY28009.1	*scsBCD*	Resistance	Suppressor for copper sensitivity operon 2. Similar operon described in *E. coli*.

YE	YP	CBY28023.1- CBY28018.1	—	Transportation	Phosphotransferase system (lactose/cellobiose).

YE	YP	CBY28059.1- CBY28057.1	*ascGFB*	Transportation	Phosphotransferase system (*β*-glycosides).

YE	YP	CBY28068.1- CBY28065.1	*yrbFE*	Transportation	ABC transporter (YrbF/E).

YE	YP	CBY28213.1- CBY28205.1	*gutQRMDBEA*	Transportation	Phosphotransferase system (glucitol/sorbitol).

YE	YP	CBY28759.1- CBY28735.1	*gldA, dhaKR, scrRBAYK*	Metabolism, transportation	Glycerol metabolism operon, phosphotransferase system (sucrose).

YE	YP	CBY29405.1- CBY29415.1	*bcsCBAFEG*	Gut colonization	Cellulose biosynthesis [21].

YE	YP	CBY29454.1- CBY29443.1	*manA, bglA, gmuD*	Transportation	Phosphotransferase system (lactose/cellobiose), maltoporin, and *β*-glucosidase

YP	YE	CAH19316.1- CAH19320.1	—	Transportation	ABC transporter (molybdate-malate).

YP	YE	CAH19585.1- CAH19591.1	—	Resistance	Methyltetrahydrofolate reduction, conserved with ter operon

YP	YE	CAH19592.1- CAH19597.1	*terZABCDE*	Resistance	Tellurite/tellurium resistance. Similar to the operon in *Y. pestis* plasmid.

YP	YE	CAH19782.1- CAH19785.1	*frwDBC, pstA*	Transportation	Phosphotransferase system (fructose).

YP	YE	CAH19879.1- CAH19896.1	*impACG, hcp, vasG, icmF*	Type VI secretion	YPTB T6SS-1, interbacterial interaction.

YP	YE	CAH20037.1- CAH20044.1	*sgbK, araD*	Transportation	ABC transporter (L-xylose), epimerase.

YP	YE	CAH20283.1- CAH20290.1	—	Unknown	CDP-diacylglycerol synthesis operon. A similar gene cluster of unknown function has been described in *E. coli*.

YP	YE	CAH20313.1- CAH20316.1	—	Transportation	ABC transporter (myoinositol), dehydrogenase.

YP	YE	CAH20560.1- CAH20567.1	*rpiA*	Transportation	ABC transporter (sugar), dehydrogenase.

YP	YE	CAH20608.1- CAH20613.1	—	Transportation	ABC transporter (iron).

YP	YE	CAH20708.1- CAH20725.1	—	Type VI secretion	Conserved area before type VI secretion system.

YP	YE	CAH20725.1- CAH20742.1	*impKL, hcp, vasGD*	Type VI secretion	YPTB T6SS-2.

YP	YE	CAH20812.1- CAH20822.1	*lidD*	Transportation	Symport.

YP	YE	CAH20875.1- CAH20884.1	*hpaRGEDFHIXBC*	Use of aromatic substances	Hpa operon.

YP	YE	CAH20923.1- CAH20928.1	—	Transportation	ABC transporter (sugar).

YP	YE	CAH21145.1- CAH21154.1	—	Transportation	ABC transporter (sorbitol).

YP	YE	CAH21162.1- CAH21172.1	—	Transportation	MFS transporter (aromatic acids).

YP	YE	CAH21251.1- CAH21255.1	*potDCBA*	Transportation	ABC transporter (polyamines).

YP	YE	CAH21445.1- CAH21448.1	*tauB*	Transportation	Transporter (taurine/sulfonate).

YP	YE	CAH21739.1- CAH21760.1	*manB, mtlK*	Transportation	MFS transporter (sugar), ABC transporter (sugar), and CRISPR repeats.

YP	YE	CAH21766.1- CAH21777.1	*gutB*	Transportation	Carnitine transporter, tartrate dehydrogenase, and ABC transporter (sorbitol).

YP	YE	CAH22045.1- CAH22050.1	*goaG*	Transportation	ABC transporter (opines/polyamines).

YP	YE	CAH22292.1- CAH22299.1	*gspLKJHI*	Type II secretion	General secretion pathway.

YP	YE	CAH22307.1- CAH22312.1	—	Growth on chondroitin sulfate	Secreted chondroitin ABC lyase.

YP	YE	CAH22317.1- CAH22328.1	*kduD2*	Transportation	Phosphotransferase system (N-acetylgalactosamine), chondro-6-sulfatase.

YP	YE	CAH22333.1- CAH22353.1	*lamb, bgaB*	Transportation	ABC transporter (maltodextrin/maltose/ribose).

YP	YE	CAH22467.1- CAH22469.1	*mglA*	Transportation	ABC transporter (sugar).

YP	YE	CAH22662.1- CAH22687.1	*yapF*	Transportation	Na+/H+-antiport, ABC transporter (sugar).

YP	YE	CAH23038.1- CAH23046.1	—	Transportation	ABC transporter (ribose), two-component system.

YE 4/O:3	YE BT 1–3, YP	CBY26503.1- CBY26517.1		Serotype O:3 antigen	dDTP-L-rhamnose biosynthesis [22, 23].

YE 4/O:3	YE BT 1–3, YP	CBY26512.1- CBY26517.1		Serotype O:3 antigen	Conserved area posterior to the O:3 antigen. Hypothetical proteins, transposon.

YE BT 2–4	YE BT 1A, 1B; YP	CBY25728.1- CBY25740.1	*aatBCAP, araC*	Resistance	Multidrug efflux system. Cluster includes 6 genes and 7 hypothetical insertion sequences.

YE BT 2–4, 1	YE BT 1A, 1B; YP	CBY29000.1- CBY29007.1	*sseDBCEBF*	Virulence, type III secretion	Type III secreting effectors and chaperones. *Salmonella* type III secretion Sse operon is involved in interaction with macrophages.

YE BT 2–4, 1A	YE BT 1B	CBY28981.1- CBY29013.1	*ysp*	Virulence, type III secretion	Not fully conserved in biotype 1A strains [24].

YE BT 2–4, 1A	YE BT 1B	CBY26978.1- CBY26985.1	*agaRZVWEFSY*	N-Acetylgalactosamine utilization	Use of intestinal mucin as a carbon source [24].

YE = *Y. enterocolitica*, YP = *Y. pseudotuberculosis*, BT = biotype(s).

## References

[1] European Food Safety Authority (2015). The European Union summary report on trends and sources of zoonoses, zoonotic agents and food-borne outbreaks in 2013. *EFSA Journal*.

[2] Bottone E. J. (1999). Yersinia enterocolitica: overview and epidemiologic correlates. *Microbes and Infection*.

[3] Achtman M., Zurth K., Morelli G., Torrea G., Guiyoule A., Carniel E. (1999). *Yersinia pestis*, the cause of plague, is a recently emerged clone of *Yersinia pseudotuberculosis*. *Proceedings of the National Academy of Sciences of the United States of America*.

[4] Reuter S., Connor T. R., Barquist L. (2014). Parallel independent evolution of pathogenicity within the genus *Yersinia*. *Proceedings of the National Academy of Sciences of the United States of America*.

[5] Wren B. W. (2003). The yersiniae—a model genus to study the rapid evolution of bacterial pathogens. *Nature Reviews: Microbiology*.

[6] Kanazawa Y., Ikemura K., Sasagawa I., Shigeno N. (1974). A case of terminal ileitis due to *Yersinia pseudotuberculosis*. *Kansenshōgaku Zasshi. The Journal of the Japanese Association for Infectious Diseases*.

[7] Tauxe R. V., Wauters G., Goossens V. (1987). *Yersinia enterocolitica* infections and pork: the missing link. *The Lancet*.

[8] Fredriksson-Ahomaa M., Wacheck S., Koenig M., Stolle A., Stephan R. (2009). Prevalence of pathogenic *Yersinia enterocolitica* and *Yersinia pseudotuberculosis* in wild boars in Switzerland. *International Journal of Food Microbiology*.

[9] Fredriksson-Ahomaa M., Hallanvuo S., Korte T., Siitonen A., Korkeala H. (2001). Correspondence of genotypes of sporadic *Yersinia enterocolitica* bioserotype 4/O:3 strains from human and porcine sources. *Epidemiology and Infection*.

[10] Fredriksson-Ahomaa M., Stolle A., Siitonen A., Korkeala H. (2006). Sporadic human *Yersinia enterocolitica* infections caused by bioserotype 4/O:3 originate mainly from pigs. *Journal of Medical Microbiology*.

[11] Virtanen S., Laukkanen-Ninios R., Martínez P. O., Siitonen A., Fredriksson-Ahomaa M., Korkeala H. (2013). Multiple-locus variable-number tandem-repeat analysis in genotyping *Yersinia enterocolitica* strains from human and porcine origins. *Journal of Clinical Microbiology*.

[12] Fredriksson-Ahomaa M., Korkeala H. (2003). Low occurrence of pathogenic *Yersinia enterocolitica* in clinical, food, and environmental samples: a methodological problem. *Clinical Microbiology Reviews*.

[13] MacDonald E., Heier B. T., Nygård K. (2012). *Yersinia enterocolitica* outbreak associated with ready-to-eat salad mix, Norway, 2011. *Emerging Infectious Diseases*.

[14] Ackers M.-L., Schoenfeld S., Markman J. (2000). An outbreak of *Yersinia enterocolitica* O:8 infections associated with pasteurized milk. *The Journal of Infectious Diseases*.

[15] Nuorti J. P., Niskanen T., Hallanvuo S. (2004). A widespread outbreak of *Yersinia pseudotuberculosis* O:3 infection from iceberg lettuce. *Journal of Infectious Diseases*.

[16] Jalava K., Hallanvuo S., Nakari U.-M. (2004). Multiple outbreaks of *Yersinia pseudotuberculosis* infections in Finland. *Journal of Clinical Microbiology*.

[17] Tertti R., Granfors K., Lehtonen O.-P., et al (1984). An outbreak of *Yersinia pseudotuberculosis* infection. *Journal of Infectious Diseases*.

[18] Rimhanen-finne R., Niskanen T., Hallanvuo S. (2009). Yersinia pseudotuberculosis causing a large outbreak associated with carrots in Finland, 2006. *Epidemiology and Infection*.

[19] Jalava K., Hakkinen M., Valkonen M. (2006). An outbreak of gastrointestinal illness and erythema nodosum from grated carrots contaminated with *Yersinia pseudotuberculosis*. *The Journal of Infectious Diseases*.

[20] Kangas S., Takkinen J., Hakkinen M. (2008). *Yersinia pseudotuberculosis* O:1 traced to raw carrots, Finland. *Emerging Infectious Diseases*.

[21] Thomson N. R., Howard S., Wren B. W. (2006). The complete genome sequence and comparative genome analysis of the high pathogenicity *Yersinia enterocolitica* strain 8081. *PLoS Genetics*.

[22] Valentin-Weigand P., Heesemann J., Dersch P. (2014). Unique virulence properties of *Yersinia enterocolitica* O:3—an emerging zoonotic pathogen using pigs as preferred reservoir host. *International Journal of Medical Microbiology*.

[23] Skurnik M., Venho R., Toivanen P., Al-Hendy A. (1995). A novel locus of *Yersinia enterocolitica* serotype O:3 involved in lipopolysaccharide outer core biosynthesis. *Molecular Microbiology*.

[24] Rakin A., Batzilla J., Garzetti D., Heesemann J. (2012). Gains and losses in *Yersinia enterocolitica* subsp. *palearctica* genomes. *Advances in Yersinia Research*.

[25] Martínez P. O., Mylona S., Drake I., Fredriksson-Ahomaa M., Korkeala H., Corry J. E. L. (2010). Wide variety of bioserotypes of enteropathogenic *Yersinia* in tonsils of English pigs at slaughter. *International Journal of Food Microbiology*.

[26] Martínez P. O., Fredriksson-Ahomaa M., Pallotti A., Rosmini R., Houf K., Korkeala H. (2011). Variation in the prevalence of enteropathogenic Yersinia in slaughter pigs from Belgium, Italy, and Spain. *Foodborne Pathogens and Disease*.

[27] Martínez P. O., Fredrlksson-Ahomaa M., Sokolova Y., Roasto M., Berzins A., Korkeala H. (2009). Prevalence of enteropathogenic *Yersinia* in Estonian, latvian, and russian (Leningrad Region) pigs. *Foodborne Pathogens and Disease*.

[28] Gierczyński R., Szych J., Rastawicki W., Wardak S., Jagielski M. (2009). Molecular characterization of human clinical isolates of *Yersinia enterocolitica* bioserotype 1B/O8 in poland: emergence and dissemination of three highly related clones. *Journal of Clinical Microbiology*.

[29] Schubert S., Bockemühl J., Brendler U., Heesemann J. (2003). First isolation of virulent *Yersinia enterocolitica* O8, biotype 1B in Germany. *European Journal of Clinical Microbiology and Infectious Diseases*.

[30] Batzilla J., Höper D., Antonenka U., Heesemann J., Rakin A. (2011). Complete genome sequence of *Yersinia enterocolitica subsp. palearctica* Serogroup O:3. *Journal of Bacteriology*.

[31] Batzilla J., Heesemann J., Rakin A. (2011). The pathogenic potential of *Yersinia enterocolitica* 1A. *International Journal of Medical Microbiology*.

[32] Carniel E., Guilvout I., Prentice M. (1996). Characterization of a large chromosomal ‘high-pathogenicity island’ in biotype 1B *Yersinia enterocolitica*. *Journal of Bacteriology*.

[33] Wang X., Li Y., Jing H. (2011). Complete genome sequence of a *Yersinia enterocolitica* ‘old world’ (3/O:9) strain and comparison with the ‘new world’ (1B/O:8) strain. *Journal of Clinical Microbiology*.

[34] Bach S., De Almeida A., Carniel E. (2000). The *Yersinia* high-pathogenicity island is present in different members of the family *Enterobacteriaceae*. *FEMS Microbiology Letters*.

[35] Ellison D. W., Lawrenz M. B., Miller V. L. (2004). Invasin and beyond: regulation of *Yersinia* virulence by RovA. *Trends in Microbiology*.

[36] Schaake J., Kronshage M., Uliczka F. (2013). Human and animal isolates of *Yersinia enterocolitica* show significant serotype-specific colonization and host-specific immune defense properties. *Infection and Immunity*.

[37] Joutsen S., Sarno E., Fredriksson-Ahomaa M., Cernela N., Stephan R. (2013). Pathogenic *Yersinia enterocolitica* O:3 isolated from a hunted wild alpine ibex. *Epidemiology and Infection*.

[38] Fukushima H., Gomyoda M. (1991). Intestinal carriage of *Yersinia pseudotuberculosis* by wild birds and mammals in Japan. *Applied and Environmental Microbiology*.

[39] Niskanen T., Fredriksson-Ahomaa M., Korkeala H. (2002). *Yersinia pseudotuberculosis* with limited genetic diversity is a common finding in tonsils of fattening pigs. *Journal of Food Protection*.

[40] R Core Team (2012). *R: A Language and Environment for Statistical Computing*.

[41] Li W., Godzik A. (2006). Cd-hit: a fast program for clustering and comparing large sets of protein or nucleotide sequences. *Bioinformatics*.

[42] Pitcher D. G., Saunders N. A., Owen R. J. (1989). Rapid extraction of bacterial genomic DNA with guanidium thiocyanate. *Letters in Applied Microbiology*.

[43] Lindström M., Hinderink K., Somervuo P. (2009). Comparative genomic hybridization analysis of two predominant Nordic group I (proteolytic) *Clostridium botulinum* type B clusters. *Applied and Environmental Microbiology*.

[44] Lahti P., Lindström M., Somervuo P., Heikinheimo A., Korkeala H. (2012). Comparative genomic hybridization analysis shows different epidemiology of chromosomal and plasmid-borne cpe-carrying *Clostridium perfringens* type A. *PLoS ONE*.

[45] Smyth G. K., Gentleman R., Carey V. J., Huber W. (2005). limma: linear models for microarray data. *Bioinformatics and Computational Biology Solutions Using R and Bioconductor*.

[46] Ritchie M. E., Silver J., Oshlack A. (2007). A comparison of background correction methods for two-colour microarrays. *Bioinformatics*.

[47] Saeed A. I., Bhagabati N. K., Braisted J. C. (2006). TM4 microarray software suite. *Methods in Enzymology*.

[48] Schwarz S., West T. E., Boyer F. (2010). Burkholderia type VI secretion systems have distinct roles in eukaryotic and bacterial cell interactions. *PLoS Pathogens*.

[49] Edgar R. C. (2004). MUSCLE: multiple sequence alignment with high accuracy and high throughput. *Nucleic Acids Research*.

[50] Gascuel O. (1997). BIONJ: an improved version of the NJ algorithm based on a simple model of sequence data. *Molecular Biology and Evolution*.

[51] Wolf Y. I., Koonin E. V. (2012). A tight link between orthologs and bidirectional best hits in bacterial and archaeal genomes. *Genome Biology and Evolution*.

[52] Russell A. B., Hood R. D., Bui N. K., Leroux M., Vollmer W., Mougous J. D. (2011). Type VI secretion delivers bacteriolytic effectors to target cells. *Nature*.

[53] Pukatzki S., Ma A. T., Sturtevant D. (2006). Identification of a conserved bacterial protein secretion system in *Vibrio cholerae* using the *Dictyostelium* host model system. *Proceedings of the National Academy of Sciences of the United States of America*.

[54] Schwarz S., Hood R. D., Mougous J. D. (2010). What is type VI secretion doing in all those bugs?. *Trends in Microbiology*.

[55] Boyer F., Fichant G., Berthod J., Vandenbrouck Y., Attree I. (2009). Dissecting the bacterial type VI secretion system by a genome wide in silico analysis: what can be learned from available microbial genomic resources?. *BMC Genomics*.

[56] Hood R. D., Singh P., Hsu F. (2010). A type VI secretion system of *Pseudomonas aeruginosa* targets a toxin to bacteria. *Cell Host & Microbe*.

[57] Robinson J. B., Telepnev M. V., Zudina I. V. (2009). Evaluation of a *Yersinia pestis* mutant impaired in a thermoregulated type VI-like secretion system in flea, macrophage and murine models. *Microbial Pathogenesis*.

[58] Mougous J. D., Cuff M. E., Raunser S. (2006). A virulence locus of *Pseudomonas aeruginosa* encodes a protein secretion apparatus. *Science*.

[59] Schell M. A., Ulrich R. L., Ribot W. J. (2007). Type VI secretion is a major virulence determinant in *Burkholderia mallei*. *Molecular Microbiology*.

[60] Shalom G., Shaw J. G., Thomas M. S. (2007). In vivo expression technology identifies a type VI secretion system locus in *Burkholderia pseudomallei*that is induced upon invasion of macrophages. *Microbiology*.

[61] Ma A. T., McAuley S., Pukatzki S., Mekalanos J. J. (2009). Translocation of a *Vibrio cholerae* type VI secretion effector requires bacterial endocytosis by host cells. *Cell Host & Microbe*.

[62] Prieto M. A., Díaz E., García J. L. (1996). Molecular characterization of the 4-hydroxyphenylacetate catabolic pathway of *Escherichia coli* W: engineering a mobile aromatic degradative cluster. *Journal of Bacteriology*.

[63] Díaz E., Ferrández A., Prieto M. A., García J. L. (2001). Biodegradation of aromatic compounds by *Escherichia coli*. *Microbiology and Molecular Biology Reviews*.

[64] Huang Y., Leming C. L., Suyemoto M., Altier C. (2007). Genome-wide screen of Salmonella genes expressed during infection in pigs, using in vivo expression technology. *Applied and Environmental Microbiology*.

