# Supplementary material for: Comparative Genomic Hybridization Analysis of Yersinia enterocolitica and Yersinia pseudotuberculosis Identifies Genetic Traits to Elucidate Their Different Ecologies
Source: Biomed Res Int. 2015 Oct 28;2015:760494. doi: 10.1155/2015/760494 (PMC4641178; doi:10.1155/2015/760494)
Supplement: Supplementary file 1 — Supplementary material includes the information of bacterial strains used for hybridization (Table S1), the information of sequences used for microarray design (Table S2) and a comparison of number of gene groups present, specific and unique for Yersinia pseudotuberculosis and Yersinia enterocolitica (Table S3). [file 760494.f1.pdf]

Table S1 *Yersinia* strains hybridized on microarrays and their name, isolation source, bioserotype and origin.

No	Species and group(s)	Strain name	Isolation source	BT/ST	Origin
1	*Y. enterocolitica*/Other biotypes & Reference strain	8081/NCTC 13174	Human	1B/O:8	Ohio, USA
2	*Y. enterocolitica*/4/O:3 & Reference strain	Y11/DSM no. 13030	Human	4/O:3	Germany
3	*Y. pseudotuberculosis* & Reference strain	IP 32953	Human	1	France*
4	*Y. enterocolitica*	Strain HV	Unknown	1B	Unknown
5	*Y. enterocolitica*/4/O:3	183 (YLUT 81.1)	Swine, tonsils	4/O:3	Finland
6	*Y. enterocolitica*/4/O:3	185 (YLUU 96.1 k)	Swine, feces	4/O:3	Finland
7	*Y. enterocolitica*/4/O:3	187 (YLUT 351.1)	Swine, tonsils	4/O:3	Finland
8	*Y. enterocolitica*/4/O:3	188 (YLUR 224.1 c)	Swine, carcass	4/O:3	Finland
9	*Y. enterocolitica*/4/O:3	191 (YLUR 96.1 k)	Swine, carcass	4/O:3	Finland
10	*Y. enterocolitica*/4/O:3	192 (YLUT 22.1 c)	Swine, tonsils	4/O:3	Finland
11	*Y. enterocolitica*/4/O:3	196 (YLUT 361.1 k)	Swine, tonsils	4/O:3	Finland
12	*Y. enterocolitica*/4/O:3	201 (YLUE 2 k)	Swine, organs	4/O:3	Finland
13	*Y. enterocolitica*/4/O:3	204 (YLUS 1.1 c)	Swine, intestines	4/O:3	Finland
14	*Y. enterocolitica*/4/O:3	206 (3.1c L)	Swine, tonsils	4/O:3	England
15	*Y. enterocolitica*/4/O:3	208 (10.1c)	Swine, tonsils	4/O:3	England
16	*Y. enterocolitica*/4/O:3	210 (4.1i)	Swine, tonsils	4/O:3	England
17	*Y. enterocolitica*/4/O:3	213 (96.2c)	Swine, tonsils	4/O:3	England
18	*Y. enterocolitica*/4/O:3	214 (YLUU 346.1 I)	Swine, feces	4/O:3	Finland
19	*Y. enterocolitica*/4/O:3	219 (144c.2)	Swine, tonsils	4/O:3	England
20	*Y. enterocolitica*/4/O:3	228 (207c.1)	Swine, tonsils	4/O:3	England
21	*Y. enterocolitica*/4/O:3	231 (RUS 6.17.3 i)	Swine, tonsils	4/O:3	Russia
22	*Y. enterocolitica*/4/O:3	232 (RUS 7.7.1 i)	Swine, tonsils	4/O:3	Russia
23	*Y. enterocolitica*/4/O:3	233 (RUS 6.11.3 i)	Swine, tonsils	4/O:3	Russia
24	*Y. enterocolitica*/4/O:3	234 (RUS 6.15.2 i)	Swine, tonsils	4/O:3	Russia
25	*Y. enterocolitica*/4/O:3	236 (RUS 2.10.1 i)	Swine, tonsils	4/O:3	Russia
26	*Y. enterocolitica*/4/O:3	238 (RUS 5.1.1 i)	Swine, tonsils	4/O:3	Russia
27	*Y. enterocolitica*/4/O:3	239 (RUS 5.10.1. i)	Swine, tonsils	4/O:3	Russia
28	*Y. enterocolitica*/4/O:3	240 (RUS 9.15.1 k)	Swine, tonsils	4/O:3	Russia
29	*Y. enterocolitica*/4/O:3	241 (RUS 9.18.1 k)	Swine, tonsils	4/O:3	Russia
30	*Y. enterocolitica*/4/O:3	244 (7.2 i (7.1))	Swine, tonsils	4/O:3	England
31	*Y. enterocolitica*/4/O:3	249 (205k.3)	Swine, tonsils	4/O:3	England
32	*Y. enterocolitica*/4/O:3	260 (YLUT 99.1 K)	Swine, tonsils	4/O:3	Finland
33	*Y. enterocolitica*/4/O:3	265 (YLUT 106.1K)	Swine, tonsils	4/O:3	Finland
34	*Y. enterocolitica*/4/O:3	266 (YLUT 107.1 K)	Swine, tonsils	4/O:3	Finland
35	*Y. enterocolitica*/4/O:3	270 (YLUT 124.4 K)	Swine, tonsils	4/O:3	Finland
36	*Y. enterocolitica*/4/O:3	272 (YLUT 129.1 K)	Swine, tonsils	4/O:3	Finland
37	*Y. enterocolitica*/4/O:3	273 (YLUT 143.2 C)	Swine, tonsils	4/O:3	Finland
38	*Y. enterocolitica*/4/O:3	278 (YLUT 168.1 K)	Swine, tonsils	4/O:3	Finland
39	*Y. enterocolitica*/4/O:3	284 (IHI 110193)	Human	4/O:3	Finland

Table S1 continues

No	Species and group(s)	Strain name	Isolation source	BT/ST	Origin
40	*Y. enterocolitica/*4/O:3	290 (IHI 111204)	Human	4/O:3	Åland
41	*Y. enterocolitica/*4/O:3	302 (IHI 111042)	Human	4/O:3	Finland
42	*Y. enterocolitica/*4/O:3	303 (IHI 111040)	Human	4/O:3	Finland
43	*Y. enterocolitica/*4/O:3	229 (E3c.1k)	Swine, tonsils	BT 4	Estonia
44	*Y. enterocolitica/*4/O:3	230 (E6d.1c)	Swine, tonsils	BT 4	Estonia
45	*Y. enterocolitica/*4/O:3	250 (E7d.2c)	Swine, tonsils	BT 4	Estonia
46	*Y. enterocolitica/*Other biotypes	218 (120.3k)	Swine, tonsils	3/O:5,27	England
47	*Y. enterocolitica/*Other biotypes	220 (126c.1)	Swine, tonsils	2/O:5,27	England
48	*Y. enterocolitica/*Other biotypes	221 (257.2i)	Swine, tonsils	2/O:9	England
49	*Y. enterocolitica/*Other biotypes	223 (133.1i)	Swine, tonsils	3/O:5,27	England
50	*Y. enterocolitica/*Other biotypes	224 (134.1i)	Swine, tonsils	3/O:5,27	England
51	*Y. enterocolitica/*Other biotypes	225 (291.1k)	Swine, tonsils	3/O:5,27	England
52	*Y. enterocolitica/*Other biotypes	254 (288.1i)	Swine, tonsils	O:9	England
53	*Y. enterocolitica/*Other biotypes	255 (290i)	Swine, tonsils	O:9	England
54	*Y. enterocolitica/*Other biotypes	257 (294.1i)	Swine, tonsils	O:9	England
55	*Y. enterocolitica/*Other biotypes	291 (LIP52 1ITC1)	Bird, droppings	1A	Finland
56	*Y. enterocolitica/*Other biotypes	292 (LIP52 3)	Bird, droppings	1A	Finland
57	*Y. enterocolitica/*Other biotypes	293 (LIP77)	Bird, droppings	1A	Finland
58	*Y. enterocolitica/*Other biotypes	296 (JUY2A 1ITC1)	Swine, carcass	1A	Finland
59	*Y. enterocolitica/*Other biotypes	297 (JUY1A ITC1)	Swine, carcass	1A	Finland
60	*Y. enterocolitica/*Other biotypes	299 (JUY4D 1ITC1)	Swine, internal organs	1A	Finland
61	*Y. enterocolitica/*Other biotypes	300 (JUYE)	Slaughterhouse surface sample	1A	Finland
62	*Y. enterocolitica/*Other biotypes	YE-ys23-m2kc	Swine, carcass	1B/O:8	Finland
63	*Y. pseudotuberculosis*	YTSKO 21.1 P1	Swine, tonsils	2/NA	Unknown
64	*Y. pseudotuberculosis*	YTSKO 21.2 K2	Swine, tonsils	2/NA	Unknown
65	*Y. pekkanenii*	ÅYV 7.1 K2	Water	2	Åland**
66	*Y. pseudotuberculosis*	LIP 65.1	Pigeon	1/O:1	Finland
67	*Y. pseudotuberculosis*	IHI 110 950	Human	1/NA	Finland
68	*Y. pseudotuberculosis*	LE 116.2	Cat	2/NA	Unknown
69	*Y. pseudotuberculosis*	470_96 Oulu	Hare	ET	Finland
70	*Y. pseudotuberculosis*	YSI 45 KS	Swine, feces	2/O:3	Finland
71	*Y. pseudotuberculosis*	YSI 85.2 K	Swine, feces	2/O:3	Finland
72	*Y. pseudotuberculosis*	YSI 116.1 R1	Swine, feces	2/NA	Finland
73	*Y. pseudotuberculosis*	YSI 120.8 S	Swine, feces	2/NA	Finland
74	*Y. pseudotuberculosis*	YSI 217 K	Slaughterhouse surface sample	2/O:3	Finland
75	*Y. pseudotuberculosis*	YSI 230 KS	Swine, feces	2/NA	Finland
76	*Y. pseudotuberculosis*	HUM B 255	Human	1/NA	Finland
77	*Y. pseudotuberculosis*	YLUS 71.4 C	Swine	2	Finland
78	*Y. pseudotuberculosis*	YLUU 77.21 K	Swine, tonsils	NA	Finland
79	*Y. pseudotuberculosis*	GNS 24_2 91	Magpie	1/O:1	Sweden
80	*Y. pseudotuberculosis*	GNS 28_2 98	Pigeon	NA/O:1	Sweden

Table S1 continues

No	Species and group	Strain name	Isolation source	BT/ST	Origin
81	*Y. pseudotuberculosis*	YEB 112	Swine	NA	England
82	*Y. pseudotuberculosis*	YEB 118	Swine	NA	England
83	*Y. pseudotuberculosis*	YEB 180	Swine	NA	England
84	*Y. pseudotuberculosis*	YEB 192	Swine	NA	England
85	*Y. pseudotuberculosis*	YEB 193	Swine	NA	England
86	*Y. pseudotuberculosis*	312k.1A	Swine	NA	England
87	*Y. pseudotuberculosis*	311.1c	Swine	NA	England
88	*Y. pseudotuberculosis*	312.1c	Swine	NA	England
89	*Y. pseudotuberculosis*	303k.2	Swine	NA	England
90	*Y. pseudotuberculosis*	21.2c	Swine	NA	England
91	*Y. pseudotuberculosis*	H5N7.12.1k	Swine	1	Belgium
92	*Y. pseudotuberculosis*	YLUS 71.3 C	Swine	3	Finland
93	*Y. pseudotuberculosis*	YLUS 71.6 C	Swine	3	Finland
94	*Y. pseudotuberculosis*	YLUU 51.1 C	Swine	3	Finland
95	*Y. pseudotuberculosis*	RUS 6.3.2k	Swine	2	Russia
96	*Y. pseudotuberculosis*	RUS 3.11.1k.2d	Swine	2	Russia
97	*Y. pseudotuberculosis*	732_98	Magpie	1/O:1	Finland
98	*Y. pseudotuberculosis*	YEB 42A	Swine	NA	England
99	*Y. pseudotuberculosis*	YSI 45.2 K	Swine, feces	2	Finland

* Presented by Elizabeth Carniel, Institut Pasteur ** Not included in the results, BT = biotype, ST = serotype, NA = Not Available

Table S2 Chromosome and plasmid sequences used in microarray design.

Genebank access	Strain and/or plasmid name	Reference article	CDS
AM286415.1[a]	*Yersinia enterocolitica subsp. enterocolitica* 8081*,* NCTC 13174	[62]	3979
AM286416.1	pYVe8081		72
FR729477.2[a]	*Yersinia enterocolitica subsp. palearctica* Y11*,* also known as DSM no. 13030	[65]	4349
FR745874.1	pYV		110
CP002246.1	*Yersinia enterocolitica subsp. palearctica 105.5R(r)*	[29]	3935
CP002247.1	105.5R (r)p plasmid		86
CP000720.1	*Yersinia pseudotuberculosis* IP 31758	[66]	4124
CP000719.1	p153kb		136
CP000718.1	p59kb		64
BX936398.1[a]	*Yersinia pseudotuberculosis* IP 32953	[67]	3901
BX936399.2	pYV		95
BX936400.1	pYptb3		42
CP001048.1	*Yersinia pseudotuberculosis* PB1/+	No related publication	4150
CP001049.1	pYP		87
CP000950. 1	*Yersinia pseudotuberculosis* YPIII	[68]	4192
AM905950.1	*Yersinia enterocolitica* plasmid pYE854	[69]	232
AF102990.1	*Yersinia enterocolitica* W22703 pYVe227	No related publication	69
AY150843.2	*Yersinia enterocolitica A127/90* plasmid	No related publication	75
AJ132618.1	*Yersinia enterocolitica* cryptic plasmid	[70]	1
FJ696405.1	*Yersinia enterocolitica* 07-*04449* plasmid	[71]	6
FJ696406.1	*Yersinia enterocolitica* 07-04449 plasmid	[71]	14
AF336309.1	*Yersinia enterocolitica* pYVe8081	[72]	67
In total	7 chromosomes, 14 plasmids		29 786

[a] Strains hybridized on microarray in this study, CDS = Coding sequence

Table S3 Numbers of gene groups that were present in all strains belonging to the given subgroup (Shared) and gene groups that were both shared and only present in strains of a given subgroup (Specific) based on hybridization results are shown in the table. Additionally, the number or specific gene groups with no known orthologs in the other species (*Y. enterocolitica* or *Y. pseudotuberculosis)* are given.

Subgroup of strains	Number of gene groups		
	Shared	Specific	With no orthologs in the other species
Y. pseudotuberculosis (n = 38)	4067	2884	906
Y. enterocolitica (n = 60)	3547	1130	448
Y. enterocolitica biotypes 2–4 (n = 51)	4496	207	Not analysed
